# Divergent climatic response patterns of radial growth in healthy and declining poplar forests in the Engebei Ecological Demonstration Zone of Ordos, China

**DOI:** 10.3389/fpls.2026.1837678

**Published:** 2026-05-11

**Authors:** Sun Xu, Li Zongshan, Zhang Xiaojuan, Bilige Siqing, Yu Ruixin, Zejiang Li, Guo Yue

**Affiliations:** 1 State Key Laboratory of Regional and Urban Ecology, Research Center for Eco-Environmental Sciences, Chinese Academy of Sciences, Beijing, China; 2 Beijing Urban Ecosystem Research Station, Research Center for Eco-Environmental Sciences, Chinese Academy of Sciences, Beijing, China; 3 Shaanxi Yan’an Forest Ecosystem National Observation and Research Station, Beijing, China; 4 National Observation and Research Station of Earth Critical Zone on the Loess Plateau in Shaanxi, Xi’an, China; 5 Ordos International Technological Innovation Center for Desertification Control, Ordos, China

**Keywords:** drought stress, growth decline, Ordos, poplar plantations, tree rings

## Abstract

**Introduction:**

Climate warming–induced extreme drought has led to substantial growth decline and extensive tree mortality in forests worldwide, with such phenomena being particularly pronounced in plantation ecosystems. This study aimed to assess the divergent climatic responses of radial growth between healthy and declining Simon poplar (*Populus simonii* Carr.) plantations.

**Methods:**

Separate ring-width chronologies were developed for healthy (1996–2024 period) and declining (1994–2024 period) Simon poplar plantations in the Engebei Ecological Demonstration Zone of Ordos, to explore their differing responses to climate factors.

**Results:**

All statistical parameters of the declining chronologies were higher than those of the healthy ones, indicating that the declining chronologies exhibited higher quality and stronger common signals, whereas the healthy chronologies were comparatively weaker. Climate–growth response analyses revealed that radial increments of declining trees displayed significantly greater sensitivity to climatic variability than healthy trees, while the basal area increment showed the opposite pattern—declining trees had markedly lower climatic sensitivity than healthy individuals. As Simon poplar trees enter the growth decline phase, drought stress exerts increasingly strong suppressive effects on radial growth, while the warming-induced stimulation of basal area increment becomes progressively weaker.

**Discussion:**

The divergent responses of radial growth and basal area increment to climate variability underscore the key climatic determinants shaping the decline of poplar plantations across the study area. Overall, this study contributes to a mechanistic understanding of climate-driven growth decline in poplar plantations of the Ordos area and provides a scientific basis for ecological restoration and sustainable management of local degraded plantations.

## 1 Introduction

The Three-North Shelterbelt Program is a national ecological initiative launched in the mid-20th century in China to address major environmental challenges, including the expansion of aridification, intensified land desertification, and frequent wind–sand hazards (National Forestry and Grassland Administration, 2018; Zhu and Zheng, 2019). The program spans the “Three North” regions—Northeast, Northwest, and North China—extendiqng ~4,500 km from east to west and ~560–1,460 km from south to north, with a total area exceeding 4 million km^2^, accounting for more than 40% of China’s land territory (Zheng et al., 2024; Feng et al., 2025). It is recognized as the world’s largest ecological restoration project in terms of spatial extent, duration, and investment, and it holds substantial ecological value for enhancing carbon sequestration, improving ecosystem services, and safeguarding ecological security in China’s drylands (Zheng and Zhu, 2017; Zhu et al., 2023). Recent assessments indicate that, over 1978–2017, the Three-North Program increased effective forest carbon sequestration by a cumulative 1.882 billion tons, with a mean annual sink of 0.047 billion tons, equivalent to ~5% of China’s industrial CO_2_ emissions over the same period (Zhu et al., 2023).

Against the backdrop of climate warming and the intensification of warm–dry conditions, widespread growth decline and tree mortality have been reported across the Three-North Shelterbelt Program in China, substantially weakening ecosystem-service provision and posing a serious threat to regional ecological security in northern China (Zhu and Zheng, 2019; Huang et al., 2020). The pronounced decline observed in the Three-North shelterbelts cannot be attributed to a single driver; rather, it reflects the combined effects of local water-resource constraints, ongoing climate change, and inappropriate afforestation strategies (Zhu and Zheng, 2019; Zhai et al., 2022). In the arid and semi-arid regions where the shelterbelts are mainly distributed, water availability is the primary limiting factor controlling vegetation growth and distribution; zonal vegetation is dominated by shrub–grass ecosystems, with only sparse forest patches occurring in favorable microhabitats (Zhu and Li, 2007). Consequently, large-scale afforestation in these regions inevitably exceeds the local vegetation water-carrying capacity and thus promotes growth decline (Zhang et al., 2016; Qi et al., 2023). Unscientific early establishment practices and insufficient post-establishment management have also contributed importantly to the observed decline (Song et al., 2009, 2017). At present, dominant planted species are largely fast-growing, water-demanding, short-lived pioneer trees (e.g., *Pinus sylvestris* var. *mongolica*, *Populus simonii*, and *Robinia pseudoacacia*), often established at excessively high stand densities that far exceed regional water constraints, thereby predisposing plantations to growth decline (Qi et al., 2023). Moreover, shelterbelts are typically characterized by high-density monocultures with low species diversity, resulting in simplified ecosystem structure, poor stability, and weak resistance to pests and diseases, which increases the likelihood of large-scale dieback (Zhu et al., 2008; Jiang et al., 2009). Finally, in areas where marked growth decline has already emerged, the lack of timely thinning and the failure to adjust community structure and species composition have further exacerbated severe deterioration of the Three-North shelterbelts (Kang et al., 2005; Song et al., 2017; Tong et al., 2019).

Tree-ring records have been widely used in paleoclimate reconstructions and in studies of forest growth responses and adaptation to climate change, owing to their broad spatial coverage, precise dating, annual resolution, long time series, and high sensitivity to climate variability (Fritts, 1966; Briffa et al., 1998; Cook et al., 2010; Zhang and Fang, 2020). Recently, increasing attention has been directed toward using tree-ring data to characterize growth-decline trends and climate sensitivity of shelterbelt forests within China’s Three-North Shelterbelt Program. For example, Liu et al. (2022a) reported that Mongolian pine (*P. sylvestris* var. *mongolica*) shelterbelts in the Bashang region of Hebei Province exhibited markedly lower growth rates in the late growth period than in the early period; drought stress was identified as the key driver of growth decline. They further showed that, with stand aging and the intensification of warm–dry conditions, the ecological resilience of tree growth to extreme drought events continued to decrease. Based on tree-ring evidence from poplar and Mongolian pine shelterbelts in Zhangwu, Fuxin (Liaoning Province), Yuan Chaofeng et al. (2025) found that growth decline was primarily manifested as a pronounced divergence between healthy and declining individuals: declining trees exhibited significantly lower radial growth rates and basal area increment than healthy trees. In addition, chronologies from declining trees showed substantially higher sensitivity to drought stress, and the authors suggested that root-system degradation and reduced water-use efficiency—leading to diminished ecological adaptability—were important causes of growth decline. Li et al. (2024) compiled a tree-ring dataset for major dominant shelterbelt species in the arid region of northern China and found that broadleaf species (poplars and black locust) displayed much stronger growth-decline signals than conifers (e.g., Chinese pine). Moreover, broadleaf species showed significantly lower resistance and recovery to extreme drought than conifers, indicating a continuing decline in drought-adaptation capacity of broadleaf shelterbelt forests in the arid zone of northern China.

In this study, we focused on a representative area in the central sector of China’s Three-North Shelterbelt Program—the Engebei Ecological Demonstration Zone in Ordos—and developed tree-ring width chronologies for healthy and declining Simon poplar (*Populus simonii*) shelterbelts. For each category, we constructed standard, residual, and basal area increment (BAI) chronologies, and compared growth-trend characteristics and climate-response sensitivity between healthy and declining trees. Our findings will advance a mechanistic understanding of the climate drivers underlying ecological degradation of the Three-North shelterbelts, and provide a scientific basis for ecological restoration and sustainable management of degraded shelterbelt forests.

## 2 Materials and methods

### 2.1 Study region

**Figure 1:**
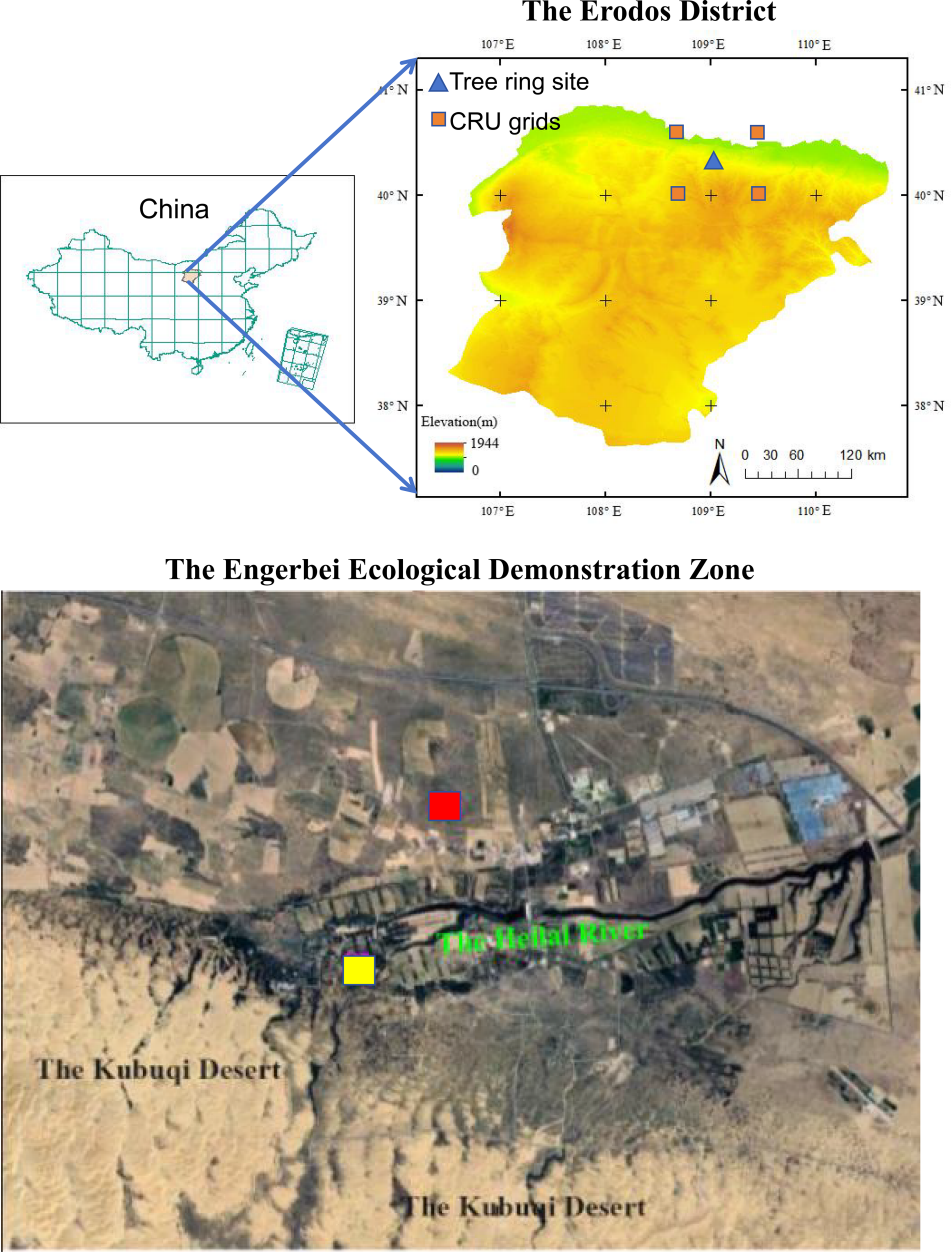
Locations of tree-ring sample sites of Simon poplar plantations in the Engerbei Ecological Demonstration Zone of the Ordos Plateau, China. The red and blue squares indicate sample sites for growth healthy and decline stand of Simon poplar plantations, respectively.

The Engebei Ecological Demonstration Zone is located on the northern margin of the Kubuqi Desert (108.7°–109.0° E, 39.3°–39.5° N) at an elevation of 1250–1450 m, and forms a sand–grass ecotone characterized by a mosaic of wind-eroded dunes and interdune depressions (Dong et al., 2026) (**Figure 1**). The regional climate is a temperate semi-arid monsoon type, with ~2850 h of sunshine per year, a mean annual temperature of 8.3 °C, an annual temperature range of ~31 °C, and a cumulative temperature above 10 °C of approximately 3000 °C; mean annual precipitation is ~330 mm and is concentrated in June–September, whereas potential evaporation reaches ~2500 mm (Sun et al., 2019). The native vegetation is dominated by desert steppe–steppe communities, with drought-tolerant shrub–herb assemblages including *Tamarix ramosissima*, *Caragana korshinskii*, *Salix cheilophila*, *Corethrodendron fruticosum*, *Artemisia desertorum*, *Stipa breviflora*, *Agropyron cristatum*, *Cleistogenes squarrosa*, *Lespedeza davurica* and *Salsola tragus* (Huang et al., 2001; Gao et al., 2017). Due to excessive grazing and land reclamation since the mid-20th century, desertification expanded and regional vegetation ecosystem services declined markedly (Niu and Li, 1992). Since the 1970s–1980s, ecological rehabilitation has been implemented in Engebei using an integrated “tree–shrub–grass + sand barriers” approach. The core afforestation species, Simon poplar, was primarily planted on fixed to semi-fixed dunes at initial densities of 2000–2500 stems ha^-1^, arranged in belts or patch plantings and protected by surrounding shrubs such as *C. korshinskii* and *S. cheilophila* (Cheng et al., 2003). After nearly four decades of succession, these plantations have developed into secondary afforested ecosystems with stand ages of 20–40 years, diameters at breast height of 10–25 cm, and canopy closure of 0.6–0.8. The understory is commonly associated with *S. cheilophila*, *C. korshinskii*, and *A. desertorum*; regional desertification has been effectively curbed, while biological soil crusts and understory biodiversity have gradually recovered (Cheng et al., 2003; Gao et al., 2017).

### 2.2 Tree-ring sampling and chronology development

**Figure 2:**
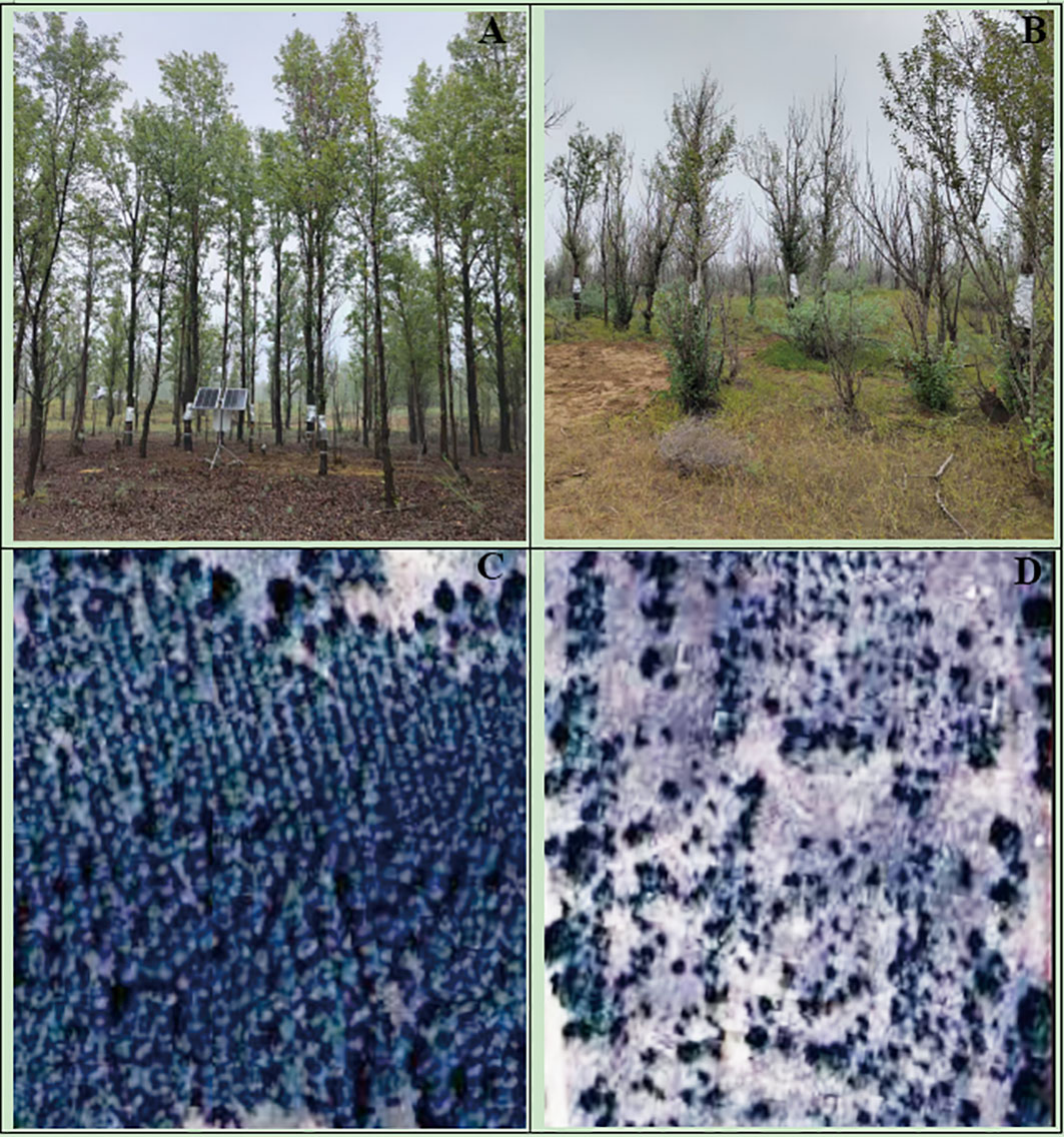
Locations of tree-ring sample sites of Simon poplar plantations in the Engerbei Ecological Demonstration Zone of the Ordos Plateau, China. **(A)** appearance of a healthy stand; **(B)** appearance of a declining stand; **(C)** landscape of a healthy stand; **(D)** landscape of a declining stand. **(A)** and **(B)** were taken in the field, while **(C)** and **(D)** were acquired from remote-sensing imagery (Jilin-1 satellite, 1.5 m spatial resolution) (Qu et al., 2022).

In the Engebei Ecological Demonstration Zone, increment cores were collected from representative stands of healthy and declining Simon poplar plantations. Tree health status was primarily identified based on external stand characteristics and high-resolution remote sensing image interpretation (**Figure 2**). Declining trees typically exhibited a crown dieback ratio of ~60–90%, together with smaller and often crooked stems, abundant and disordered branching, and a distinctive “small old tree” appearance; in contrast, healthy trees showed little or no apparent crown dieback, had larger and straighter stems, and generally fewer branches (Zhu and Li, 2007). In addition, 1.5−meter resolution optical remote sensing images from the Jilin−1 satellite acquired during the vegetation growing seasons of 2024–2025 were obtained for the study area. Visual interpretation based on canopy color, coverage, shadow characteristics, and other features was used to assist in delineating the boundaries between healthy and declining stands (Qu et al., 2022). Cores were extracted at approximately 1.3 m above ground (breast height) using a 5.15-mm diameter increment borer; depending on site conditions, coring height and aspect varied slightly. Each core was placed in a labeled plastic tube and sealed for storage (Fritts, 1966). To maximize the number of individuals sampled across a relatively large spatial extent under a comparable sampling effort, we collected 40 cores from healthy trees and 40 cores from declining trees (80 cores in total), taking only one core per tree. This sampling strategy minimizes potential bias in chronology development arising from individual-level variability.

After the samples were returned to the laboratory, all increment cores were processed following the standard procedures recommended by the Laboratory of Tree-Ring Research, University of Arizona (Stokes and Smiley, 1968). Briefly, cores were air-dried, mounted with white glue, and progressively sanded and polished using 240-, 400-, 600-, 800-, and 1000-grit sandpaper until ring boundaries were clearly visible and suitable for dendrochronological analysis. Tree-ring widths were then measured to a precision of 0.001 mm using a LINTAB ring-width measuring system manufactured by Rinntech (Frank Rinn). To ensure dating and measurement accuracy, cross-dating and measurement series were checked using the COFECHA program (Grissino-Mayer, 2001). Cores exhibiting irregular growth, decay, or breakage, and those failing to reach the 95% confidence level in correlation with the master series were excluded. Ultimately, 32 and 33 cores were retained for healthy and declining trees, respectively (**Table 1**). The standardization of tree-ring width series in ARSTAN was performed based on the growth curve morphology of each sample core. For cores exhibiting significant age-related growth trends, a negative exponential function was prioritized to eliminate age progression effects. For cores with less pronounced low-frequency non-climatic noise trends, a spline smoothing function with specific parameters (e.g., 50% cutoff frequency and 30-year rigidity) was applied (Cook and Kairiukstis, 1990). This methodology effectively removed non-climatic signals arising from genetic factors, competitive pressures, or environmental disturbances (such as competitive release/suppression effects), thereby yielding standardized tree-ring chronology sequences. Standardized series were then combined using a biweight robust mean to produce both standard and residual ring-width chronologies (Cook and Kairiukstis, 1990). The standard chronology is generally considered to preserve low-frequency variability in growth as much as possible, whereas the residual chronology, after removal of autocorrelation via an autoregressive (AR) model, primarily retains high-frequency growth variability (Fritts, 2001).

**Table 1:** Site information and statistics for standard tree-ring chronologies of growth healthy and decline planted Simon poplar forests in the Engerbei Ecological Demonstration Zone of the Ordos Plateau, China.

Type	Growth status	Location	Elevation(m)	Time length	Age	Samples	MS	SD	AC1	Rbar	SNR	EPS
Tree ring	Growth healthy	109.33°E,40.3°N	1230	1996–2024	29	32	0.384	0.291	0.234	0.322	16.66	0.943
Tree ring	Growth decline	109.33°E,40.3°N	1230	1994–2024	31	33	0.414	0.493	0.642	0.437	27.16	0.945

MS, Mean sensitivity; SD, Standard deviation; AC1, first-order autocorrelation; Rbar, Mean series inter-correlation; SNR, Signal-to-noise-ratio; EPS, Express population signal.

In addition, when annual tree-ring width remains relatively stable, basal area increment (BAI) may still increase because stem radius continues to expand over time (Liu et al., 2022b). Therefore, we also developed BAI chronologies (Basal Area Increment). Because BAI chronologies can reflect patterns of cumulative wood-volume accumulation, they may capture variability that differs from that contained in conventional ring-width chronologies (Yuan et al., 2025). BAI chronologies were computed in R using the dplR package (Bunn, 2008). Annual basal area increment was calculated as:


$$BAI=\pi (R_{n}^{2}-R_{n-1}^{2})$$

Where *R_n_* is the stem radius in year n, and *R_n-1_* is the stem radius in year n−1.

### 2.3 Data analysis

Because the sampling sites are far from meteorological stations and exhibit substantial differences in elevation and geomorphic setting, climate data were obtained from the CRU TS v4.09 global gridded dataset (0.5° resolution) (Harris et al., 2014). Monthly climate series for the period 1950–2024 were extracted from the four CRU grid cells closest to the sampling locations. Five variables were used: monthly mean temperature, monthly maximum temperature, monthly minimum temperature, precipitation, and the Standardized Precipitation Evapotranspiration Index (SPEI). For each variable, the regional climate series was calculated as the average of the four extracted grid-cell series. To account for potential lagged effects of antecedent climate on current-year growth, we correlated the standard ring-width chronology with climate variables from June of the previous year through October of the current year. SPEI is a multi-timescale drought index derived from the standardized difference between precipitation and potential evapotranspiration (Vicente-Serrano et al., 2010); it integrates the combined effects of temperature increase and precipitation variability on drought severity and its spatiotemporal evolution and has been widely applied in dendroclimatological and ecological studies (Cook et al., 2010). Correlation analyses between tree-ring data and climate variables were conducted using DendroClim2002 (Biondi and Waikul, 2004).

## 3 Results

### 3.1 Statistical characteristics of the tree-ring chronologies

According to the statistical characteristics of the chronologies (**Table 1**), all major parameters of the declining chronology—including standard deviation (SD), mean sensitivity (MS), standard deviation, first-order autocorrelation (AC1), mean series inter-correlation (Rbar), and signal-to-noise ratio (SNR)—were higher (SD = 0.493, MS = 0.414, AC1 = 0.642, Rbar=0.437, SNR = 27.16) than those of the healthy chronology (SD = 0.291, MS = 0.384, AC1 = 0.234, Rbar=0.322, SNR = 16.66). This indicates that the declining chronology has overall higher statistical quality and exhibits greater interannual growth variability as well as higher sensitivity to external environmental forcing than the healthy chronology. In addition, the expressed population signal (EPS) of both the healthy (EPS = 0.943) and declining (EPS = 0.945) chronologies approached the commonly used threshold of 0.85 (Wigley et al., 1984), suggesting that the developed chronologies are sufficiently representative of population-level growth variability of Simon poplar in the study area, and that the tree-ring data used here are suitable for dendroecological analyses.

### 3.2 Variability in climate variables and tree-ring chronologies

**Figure 3:**
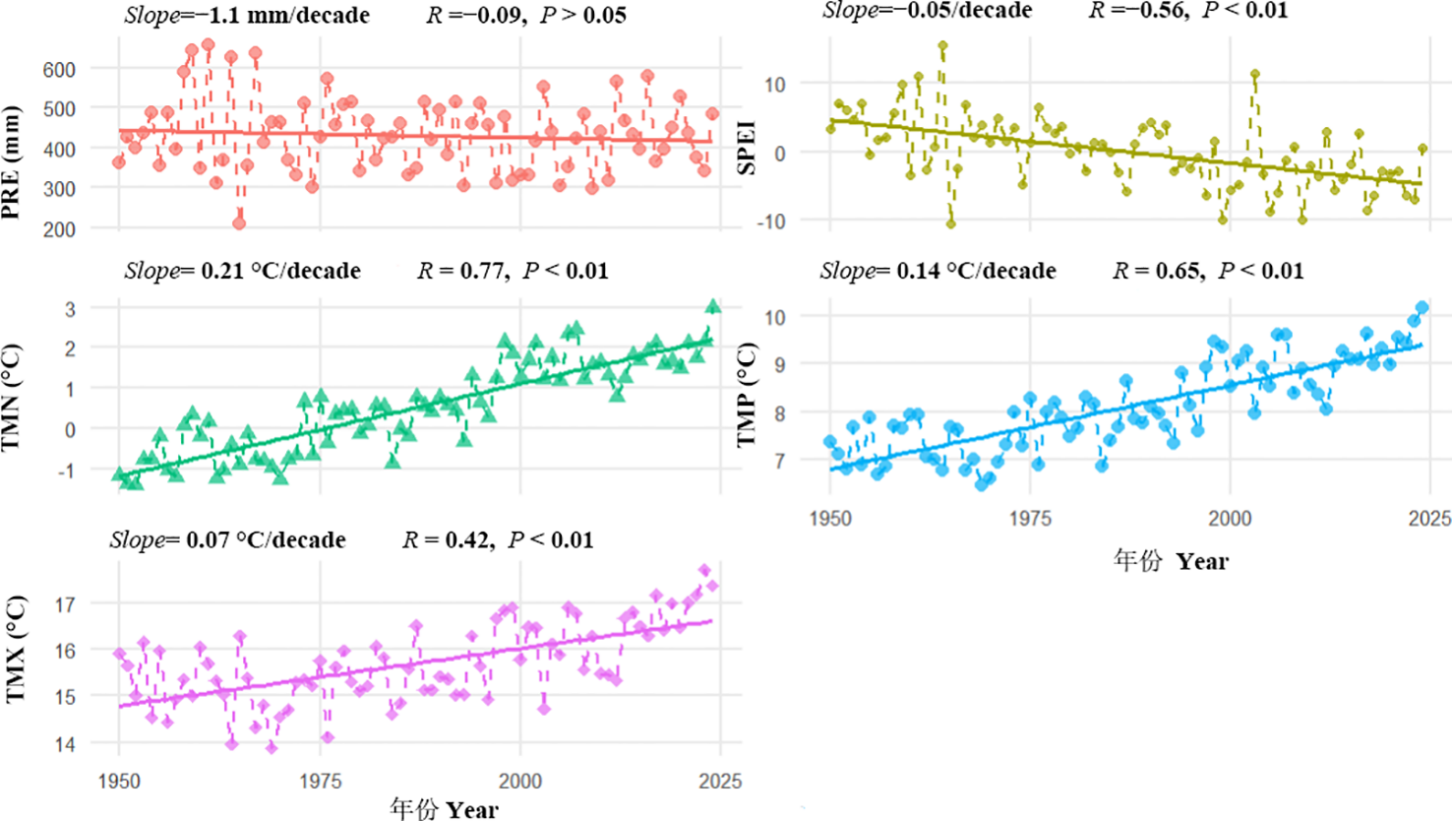
Changing trends of climatic variables during the past decades in the Engerbei Ecological Demonstration Zone of the Ordos Plateau, China. PRE, annual precipitation; SPEI, annual Standardized Precipitation Evapotranspiration Index; TMP, annual mean Temperature; TMX, annual maximum Temperature; TMN, annual minimum Temperature; Slope, decadal changing rate; R, correlation coeficient.

Based on CRU gridded climate data nearest from the sampling sites (**Figure 3**), all temperature indices exhibited significant increasing trends over the period 1950–2024. The strongest warming was observed for minimum temperature (*slope* = +0.21 °C decade^-1^, *R* = 0.77, *P* < 0.01), followed by mean temperature (*slope* = +0.14 °C decade^-1^, *R* = 0.65, *P* < 0.01), whereas maximum temperature showed the smallest increase (*slope* = +0.07 °C decade^-1^, *R* = 0.42, *P* < 0.01). In contrast, the SPEI drought index declined significantly during 1950–2024 (*slope* = −0.04 decade^-1^, *R* = −0.56, *P* < 0.01), indicating an overall intensification of drought conditions. Precipitation showed a slight decreasing trend (*slope* = −1.1 mm decade^-1^, *R* = −0.09, *P* > 0.01), but this trend was not statistically significant. Collectively, these results suggest that the study area has experienced an overall shift toward warmer and drier conditions over recent decades, driven primarily by pronounced warming rather than by substantial changes in precipitation.

**Figure 4:**
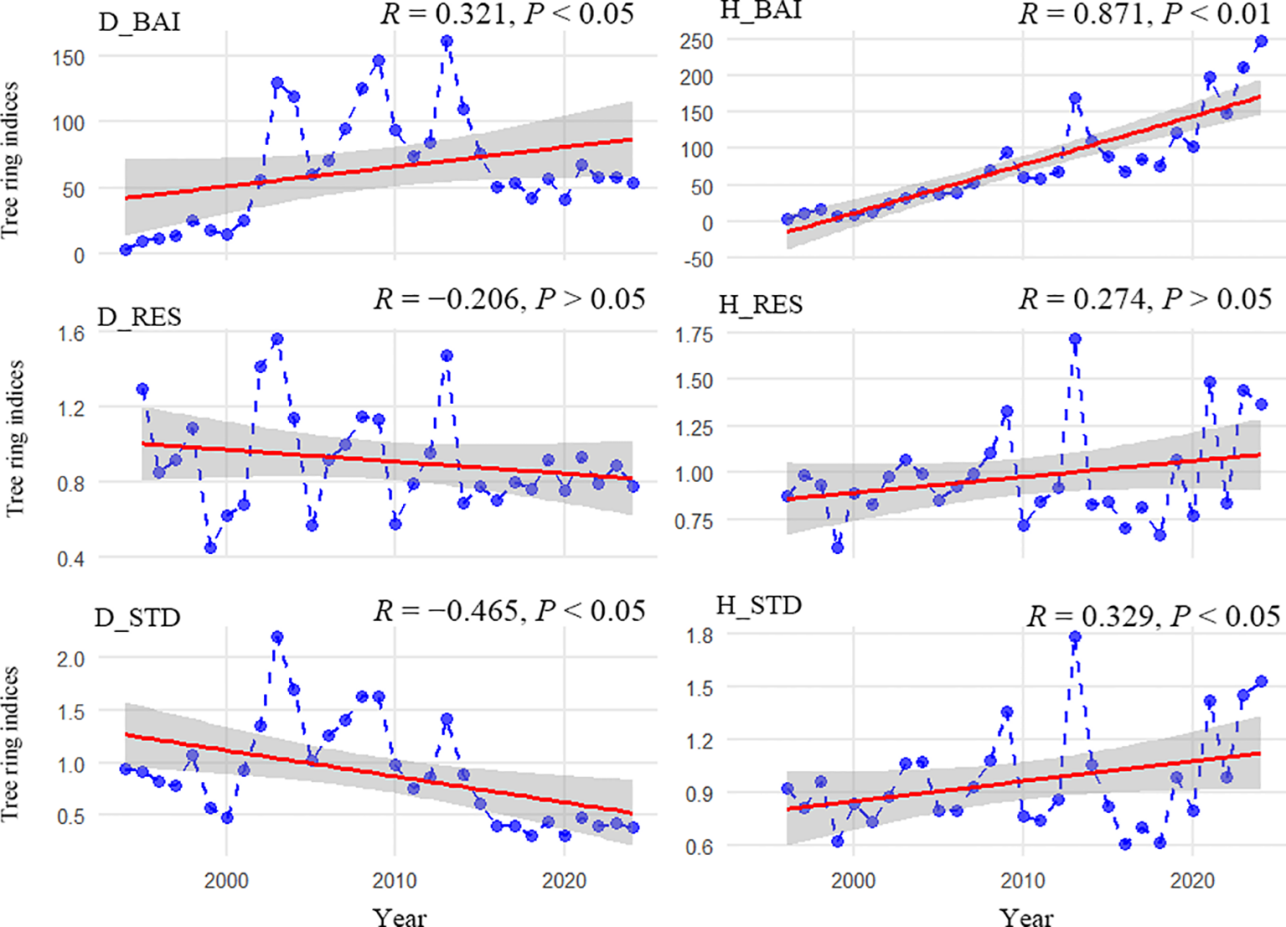
Changing trends of Simon poplar chronologies during the past decades in the Engerbei Ecological Demonstration Zone f the Ordos Plateau, China. D_BAI, H_BAI, D_RES, H_RES, D_STD, H_STD represent BAI (Basal Area Increment), residual and standard chronologies for growth healthy and decline stands, respectively.

Based on the temporal evolution of interannual variability in the chronologies (**Figure 4**), both the standard and residual chronologies of healthy Simon poplar showed significant increasing growth trends (*R* = 0.274–0.329, *P* < 0.01), whereas the corresponding declining chronologies exhibited significant decreasing trends (*R* = −0.206 to −0.465, *P* < 0.01). In addition, the basal area increment (BAI) chronologies for both healthy and declining trees showed upward trends; however, the increase was markedly stronger in the healthy chronology (*R* = 0.871, *P* < 0.01) than in the declining chronology (*R* = 0.321, *P* < 0.01).

### 3.3 Climate–growth relationships of the tree-ring chronologies

**Figure 5:**
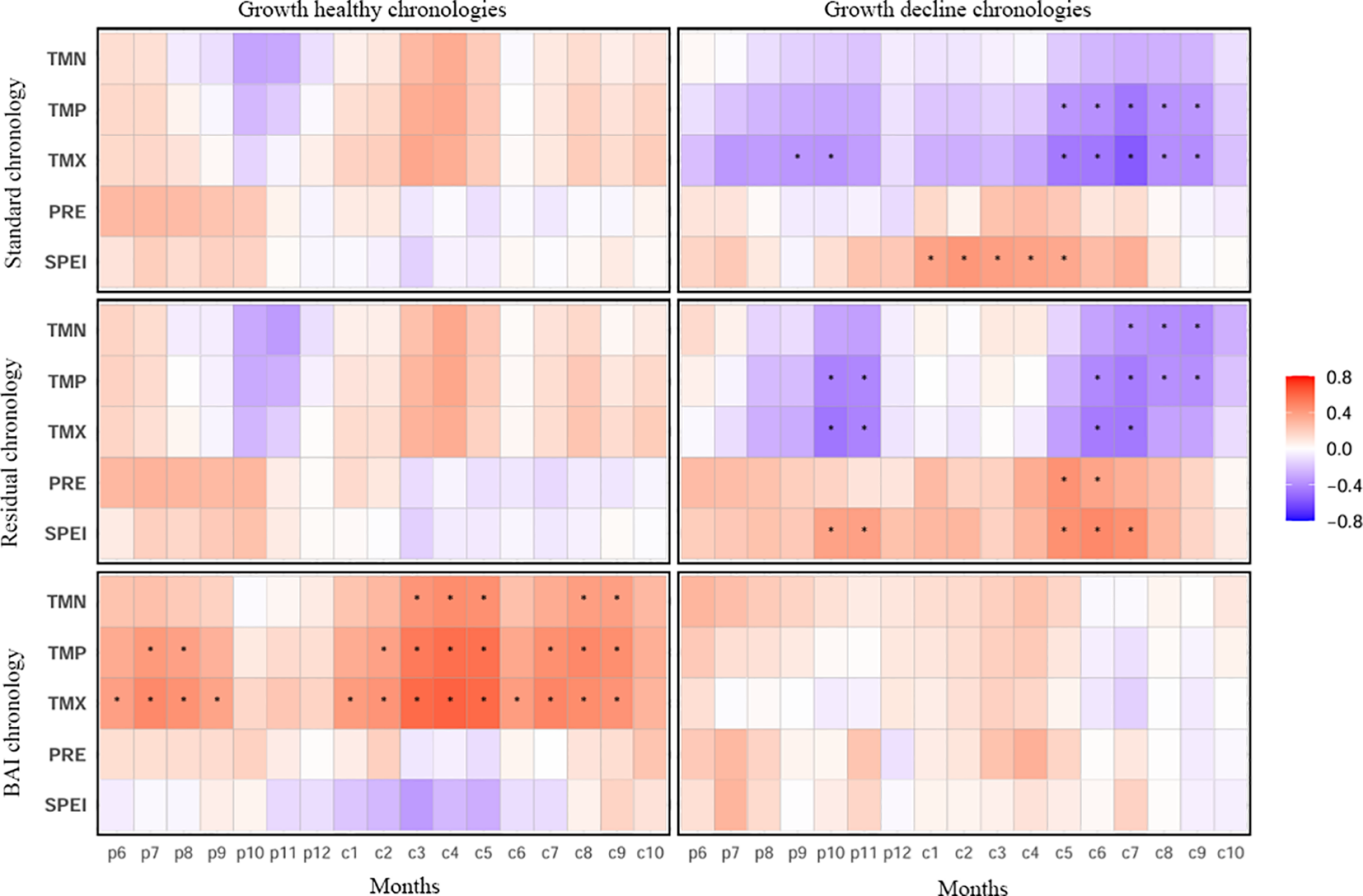
Correlations between Simon poplar chronologies in the Engerbei Ecological Demonstration Zone with monthly climate factors from previous year June to current year October. TMN, Monthly Mean Minimum Temperature; TMP, monthly mean temperature; TMX, monthly mean maximum temperature; PRE, monthly precipitation; SPEI, standardized precipitation evapotranspiration index. * indicates the correlation coefficient was significant at the 95% confidence level.

Correlation analyses between the chronologies and climate variables (**Figure 5**) revealed pronounced differences in climate sensitivity between healthy and declining Simon poplar trees for both the standard and residual chronologies. Specifically, the healthy chronologies showed generally positive correlations with the temperature variables, but the relationships were weak and mostly not statistically significant. In contrast, the declining chronologies exhibited generally negative correlations with temperature, with stronger relationships that were statistically significant in most cases, concentrated mainly in spring to summer of the current year and autumn of the previous year. Moreover, the healthy chronologies showed overall weak positive correlations with precipitation and the SPEI drought index, whereas the declining chronologies displayed markedly stronger positive correlations with precipitation and SPEI, with significant effects primarily occurring in summer of the current year and autumn of the previous year.

The BAI chronologies of healthy and declining Simon poplar also showed clearly contrasting climate-response patterns. Specifically, the healthy BAI chronology exhibited consistently strong positive correlations with all temperature variables, and the relationships were statistically significant. By contrast, although the declining BAI chronology was generally also positively correlated with temperature, the correlations were notably weaker and mostly not significant. In addition, the healthy BAI chronology showed overall weak negative correlations with precipitation and the SPEI drought index, whereas the declining BAI chronology showed overall weak positive correlations with precipitation and SPEI; however, neither set of relationships reached statistical significance.

### 3.4 Climate-response sensitivity of the tree-ring chronologies

Based on the sensitivity metrics describing climate–growth relationships (**Table 2**), the standard and residual chronologies of declining Simon poplar exhibited markedly higher climate-response sensitivity than those of healthy trees. The largest increases in sensitivity were observed for maximum temperature, precipitation, and the SPEI drought index, whereas the increases in sensitivity to mean and minimum temperature were comparatively smaller. In addition, the declining BAI chronology showed substantially enhanced sensitivity to the temperature variables relative to the healthy BAI chronology, while its sensitivity to precipitation and SPEI tended to decrease.

**Table 2:** The comparison of response sensitivity to climatic variables of the chronologies for growth healthy and decline planted Simon poplar forests in the Engerbei Ecological Demonstration Zone of the Ordos Plateau, China.

Chronology type	Growth status	TMP	△TMP	TMN	△TMN	TMX	△TMX	PRE	△PRE	SPEI	△PDSI
Standard chronology	Healthy	0.005 ± 0.148^a^	↑0.132	−0.031 ± 0.145^a^	↑0.03	0.046 ± 0.143^a^	↑0.304	0.025 ± 0.055^a^	↑0.12	−0.015 ± 0.103^a^	↑0.236
	Decline	−0.127 ± 0.114^a^		−0.034 ± 0.072^a^		−0.258 ± 0.128^a^		0.123 ± 0.122^a^		0.221 ± 0.162^b^	
Residual chronology	Healthy	−0.008 ± 0.139^a^	↑0.157	−0.025 ± 0.145^a^	↑0.05	0.03 ± 0.13^a^	↑0.222	0.004 ± 0.097^a^	↑0.297	−0.034 ± 0.086^a^	↑0.314
	Decline	−0.149 ± 0.244^a^		−0.077 ± 0.247^a^		−0.192 ± 0.221^a^		0.293 ± 0.119^b^		0.28 ± 0.162^b^	
BAI chronology	Healthy	0.448 ± 0.091^a^	↓0.384	0.357 ± 0.082^a^	↓0.255	0.475 ± 0.097^a^	↓0.466	0.055 ± 0.119^a^	↑0.04	−0.119 ± 0.178^a^	↑0.165
	Decline	0.064 ± 0.108^b^		0.102 ± 0.095^b^		0.029 ± 0.114^b^		0.095 ± 0.123^a^		0.046 ± 0.071^a^	

Different letters in the same column indicate a Dunn’s test with Bonferroni correction (*p* < 0.05). TMP, Monthly Mean Temperature; TMX, Monthly Mean Maximum Temperature; TMN, Monthly Mean Minimum Temperature; PRE, Monthly Precipitation; SPEI, Standardized Precipitation Evapotranspiration Index. △TMP, △TMN, △TMX, △PRE, △SPEI indicate the changing amplitude of climatic sensitivity for TMP, TMN, TMX, PRE and SPEI, respectively. All the correlation values in this table were averaged from the monthly correlation values during the analyzed period from June in prior year and October in current year. Upward arrow (↑) indicates the increase in correlation coefficient of the healthy tree-ring chronology relative to the declining chronology; downward arrow (↓) indicates the decrease in correlation coefficient of the declining chronology relative to the healthy chronology.

### 3.5 Multiple linear models of climate controls on tree growth

Because healthy and declining Simon poplar chronologies exhibited clearly different sensitivities to thermal variables (temperature indices) and moisture variables (precipitation and the SPEI drought index) (**Figure 5**; **Table 2**), we developed separate multiple regression models for healthy and declining chronologies using thermal and moisture predictors (**Tables 3**), with the aim of quantifying the explanatory power of climate variables for interannual variability in each chronology type. Results from the models based on thermal variables (**Table 3**) showed that temperature explained substantially more variance in the declining standard and residual chronologies than in the healthy chronologies, whereas the variance explained by temperature for the declining BAI chronology was markedly lower than that for the healthy BAI chronology. Results from the models based on moisture variables (**Table 3**) indicated that moisture predictors explained substantially more variance in the declining standard and residual chronologies than in the healthy chronologies. In contrast, moisture predictors explained relatively little variance in both healthy and declining BAI chronologies, although the explained variance was comparatively higher for the healthy BAI chronology.

**Table 3:** Multiple linear regression models of thermal-related factors for growth healthy and decline planted Simon poplar forests in the Engerbei Ecological Demonstration Zone of the Ordos Plateau, China.

Chronology type	Growth status	Linear regression model	*R^2^*	adjusted *R^2^*	*P*
Standard chronology	Healthy	Y = 0.983×TMP − 0.528×TMN − 0.362×TMX− 1.084	0.074	0.056	> 0.05
	Decline	Y =− 5.605×TMP^*^ + 3.181×TMN^*^ +2.261×TMX + 9.084	0.29	0.265	< 0.01
Residual chronology	Healthy	Y = −0.035×TMP − 0.019×TMN + 0.131×TMX− 0.823	0.069	0.036	> 0.05
	Decline	Y = −2.485×TMP + 1.262×TMN^*^ + 1.145×TMX + 2.448	0.195	0.154	< 0.05
BAI chronology	Healthy	Y = 1107×TMP^*^ − 554.4×TMN^*^ − 501.1×TMX− 765.1	0.33	0.265	< 0.01
	Decline	Y = 96.41×TMP^*^ − 16.33×TMN^*^ − 73.9×TMX+ 434.16	0.09	0.059	> 0.01

The multiple linear regression model uses linear regression methods to explain the degree of linear fit and the explained variance of the dependent variable (Y) by multiple independent variables (X_1_, X_2_, ···, X_i_). *indicated the correlations between the independent and dependent variables reaching a 95% confidence level. *R^2^* represents the explained variance, while Adjusted *R^2^* accounts for the degrees of freedom in the explained variance. TMP, TMN, TMX indicated annual mean values of monthly mean, minimum and maximum temperatures, respectively.

## 4 Discussion

### 4.1 Differences in interannual growth trends between healthy and declining Simon poplar

This study revealed pronounced differences in radial growth dynamics between healthy and declining Simon poplar in the Ordos Plateau of China (**Figure 4**). Specifically, the radial growth rates of declining trees (standard and residual chronologies) showed a clear downward trend over time, indicating evident growth decline, whereas the radial growth rates of healthy trees increased over time without an apparent decline signal. Moreover, although the radial growth rate of declining trees decreased, their cumulative stemwood and biomass accumulation (as reflected by the BAI chronology) continued to increase over time (**Figure 4**), but the magnitude of this increase was substantially weaker than that observed in healthy trees. Under drought stress, declining trees may maintain wood-volume and biomass accumulation through physiological adjustments such as extending the effective growing period and altering the phloem-to-xylem allocation ratio (Adams et al., 2017; Anderegg et al., 2022). However, as crown dieback and leaf area index further decrease, photosynthesis and carbon assimilation become increasingly constrained, which would inevitably cause the rate of volume and biomass accumulation to level off and ultimately lead to a turning point at which both radial growth and cumulative volume/biomass begin to decline synchronously (Allen et al., 2010; Choat et al., 2012). In contrast, healthy trees, owing to deeper root distribution and higher sapwood hydraulic conductivity, can sustain relatively high stomatal conductance and photosynthetic rates under drought, allocating more assimilates to secondary growth and thereby achieving concurrent increases in radial growth and volume/biomass accumulation (Greenwood et al., 2017; Gazol et al., 2025).

### 4.2 Differences in climate–growth responses between healthy and declining Simon poplar

We found that the chronology quality of declining trees was higher than that of healthy trees (**Table 1**), and that the radial-growth chronologies (standard and residual) of declining trees showed stronger correlations with climate variables and higher climate-response sensitivity than those of healthy trees (**Table 2**; **Figure 5**). In addition, multiple linear regression models based on thermal and moisture variables consistently indicated that climate factors explained substantially more of the overall variance in radial growth of declining trees than in healthy trees (**Table 3**). Collectively, these results demonstrate that radial growth of declining Simon poplar is generally more sensitive to external climatic conditions than that of healthy trees, implying an amplification of climatic-stress effects in declining individuals. Declining trees often have reduced sapwood water-transport capacity and may experience pronounced xylem embolism at relatively modest water-potential thresholds (Choat et al., 2012; Sperry and Love, 2015). Such greater hydraulic vulnerability increases the likelihood of hydraulic transport impairment (hydraulic failure), thereby enhancing sensitivity to environmental fluctuations (Adams et al., 2017; Comtet et al., 2017). Furthermore, declining trees typically exhibit lower concentrations of non-structural carbohydrates (NSC) in both crowns and roots, and limited carbon reserves are preferentially allocated to maintaining basic metabolic function rather than to radial increment (Gauthey et al., 2022; Lens et al., 2022). Insufficient soluble carbon reserves and regulatory capacity can therefore intensify carbon starvation, which is another important reason for heightened environmental sensitivity (Li et al., 2022; Joshi et al., 2022).

Our results further show that climate responses of declining trees are characterized by a pronounced inhibitory effect of temperature on radial growth, whereas moisture variables exert a clear positive effect. This pattern indicates that drought stress associated with recent warm–dry conditions is the dominant factor controlling radial growth in declining trees, ultimately leading to evident growth decline. In contrast, for healthy trees, both temperature and moisture variables exerted a generally positive influence on radial growth, suggesting that recent warm–dry conditions have, on balance, promoted radial growth in healthy individuals and thus supported a sustained increasing growth trend. Under continued warming and enhanced evaporative demand, rapid depletion of available soil water can impose chronic water-deficit pressure on the hydraulic system, reducing leaf photosynthetic rates and cambial efficiency and thereby contributing to pronounced radial-growth decline in the declining trees (del Campo et al., 2020; Dong et al., 2026). By comparison, the positive responses of healthy trees to both temperature and moisture likely reflect their higher water-use efficiency and more functional hydraulic architecture (Atkin and Tjoelker, 2003). Long-term adjustment of vessel diameter, vessel density, and physiological activity may confer a larger hydraulic safety margin, enabling maintenance of physiological function before drought stress becomes severe (Comtet et al., 2017). In addition, greater hydraulic stability in healthy trees can help sustain cambial cell division and secondary-wall thickening during xylogenesis, ultimately supporting higher radial growth rates (Rossi et al., 2006; Li et al., 2019).

Notably, compared to mean monthly temperature, maximum temperature (TMX) exerts a more pronounced inhibitory effect on the radial growth of declining trees (**Table 2**; **Figure 5**). This may be attributed to the fact that elevated daily maximum temperatures increase atmospheric vapor pressure deficit (VPD), exacerbating leaf water imbalance and the risk of xylem embolism (Allen et al., 2010; Adams et al., 2017). The narrower hydraulic safety margins of declining trees may render them more susceptible to conductive blockages under high VPD conditions, resulting in reduced allocation of photosynthetic products to secondary growth (Atkin and Tjoelker, 2003; Anderegg et al., 2022).

We also found that interannual volume and biomass dynamics of healthy trees (BAI chronology) were markedly more sensitive to climate than those of declining trees (**Table 3**; **Figure 5**), most notably through strong positive relationships between temperature indices and annual BAI in healthy trees, whereas these relationships weakened substantially in declining trees. These results indicate that recent warming has strongly promoted volume and biomass accumulation in healthy trees, whereas the stimulatory effect of warming on accumulation in declining trees is relatively limited. A warmer climate can extend the growing season of healthy trees, providing a longer period for photosynthesis and thereby increasing carbon assimilation and biomass accumulation (Körner, 2015). Moreover, intact root and leaf function in healthy trees facilitates efficient use of water and nutrients; under warming, enhanced transpiration may promote water and nutrient uptake and transport, further supporting increases in volume and biomass (Linderholm and Linderholm, 2004). Additionally, cell division and expansion may be more active in warm conditions, accelerating cambial activity and increasing annual ring formation, which can translate into greater volume and biomass accumulation (Deslauriers and Rossi, 2019). In contrast, impaired root and leaf function in declining trees reduces water and nutrient uptake capacity; under warming, increased transpiration may aggravate water deficits, thereby constraining volume and biomass accumulation (Basnet et al., 2024). Declining trees also tend to exhibit weakened cambial activity, resulting in limited increases in ring formation and a slower rate of volume/biomass accumulation (Antonucci et al., 2019). Furthermore, reduced antioxidant capacity may limit the ability of declining trees to cope with warming-induced oxidative stress, causing cellular membrane damage and deterioration of photosynthetic organs, which further restricts continued increases in biomass accumulation (Perez-de-Lis et al., 2017).

Additionally, the BAI chronology of declining trees exhibited a markedly reduced sensitivity to moisture conditions, whereas the BAI chronology of healthy stands demonstrated high responsiveness to moisture availability. Improved water supply may not be effectively utilized by severely declining individuals, as these trees may have lost most of their functional root systems or possess substantial amounts of non-functional xylem, thereby limiting water uptake and transport efficiency (Gauthey et al., 2022; Basnet et al., 2024). Under carbon starvation conditions, even when moisture conditions improve, carbon allocation mechanisms tend to prioritize sustaining vital metabolic activities over wood accumulation; consequently, the BAI chronology shows no significant response or even exhibits a declining trend under improved moisture conditions (Greenwood et al., 2017; Gazol et al., 2025).

### 4.3 Pathways for structural transformation and functional enhancement of degraded Simon poplar shelterbelts

The strong divergence between healthy and declining Simon poplar plantations in growth trends and climate responses in the Ordos Plateau provides a scientific basis for community-structure transformation and ecological-function enhancement of degraded shelterbelt forests. To address structural deficiencies in degraded plantations, multi-scale interventions are required. At the individual-tree scale, healthy Simon poplar should be prioritized as seed trees or structural “framework” individuals; thinning should target declining trees to reduce stand density and optimize light allocation. Meanwhile, deep-rooted native species such as Mongolian pine can be introduced to build mixed stands and improve water-use efficiency (Kang et al., 2005; Song et al., 2017). At the community scale, strip or patch thinning can be implemented to retain healthy individuals and interplant drought-tolerant shrubs such as *S. cheilophila*, thereby forming a tree–shrub–grass composite structure and enhancing stand resistance to stress (Zhu and Zheng, 2019). In addition, assisted regeneration can be promoted by planting stress-tolerant seedlings in canopy gaps to accelerate generational turnover and reduce risks associated with genetic deterioration in single-species systems (West, 2024). Collectively, these measures can increase hydraulic safety margins and help restored stands maintain higher growth rates under warm–dry conditions (Choat et al., 2012; Anderegg et al., 2022). Finally, our results also indicate that radial growth and cumulative volume/biomass exhibit distinct temporal trajectories and climate drivers, implying that relying solely on volume or biomass accumulation in shelterbelt health assessments may mask early signals of growth decline. Therefore, an integrated diagnostic framework should incorporate radial growth rate, tree-ring anatomical traits, stand structure, and drought-response sensitivity, coupled with timely adaptive management (e.g., density regulation, soil improvement, and mixed-species planting) to delay or reverse growth decline in arid-region plantations (Jiang et al., 2009; Stavi et al., 2023).

## 5 Conclusion

In this study, we selected representative Simon poplar shelterbelt plantations in the Ordos Plateau of China and developed ring-width chronologies for healthy and declining trees, followed by analyses of growth trends and climate-response sensitivity differences in the healthy and declining trees. The results show that declining trees exhibited a pronounced decreasing trend in radial growth, while cumulative stemwood/biomass (BAI) still increased over time. In contrast, healthy trees showed increasing trends in both radial growth and cumulative accumulation. Chronologies from declining trees showed higher statistical quality and greater climate-response sensitivity, and recent warm–dry conditions exerted an overall inhibitory effect on their radial growth. Healthy-tree chronologies showed lower statistical quality and weaker climate-response sensitivity, and recent warm–dry conditions exerted an overall positive influence on their radial growth. In addition, climate sensitivity of cumulative accumulation (BAI) was substantially higher in healthy than in declining trees, indicating that recent warming has strongly promoted volume/biomass accumulation in healthy trees, whereas this stimulatory effect was markedly weakened in declining trees. Overall, drought stress driven by recent warming-induced warm–dry conditions is the primary trigger of growth decline in Simon poplar shelterbelts in the Ordos Plateau of China. These findings provide a scientific basis for ecological restoration and sustainable management of degraded shelterbelt plantations in drylands of China.

## Data Availability

The original contributions presented in the study are included in the article/supplementary material. Further inquiries can be directed to the corresponding author.
